# Oral-Genital Contact and the Meaning of “Had Sex”: The Role of Social Desirability

**DOI:** 10.1007/s10508-021-02220-4

**Published:** 2022-02-07

**Authors:** Jessica Den Haese, Bruce M. King

**Affiliations:** 1 Honors College, Clemson University, Clemson, SC 29634 USA; 2 Department of Psychology, Clemson University, 418 Brackett Hall, Clemson, SC 29634 USA

**Keywords:** Sexual behavior, Oral-genital sex, Social desirability, Technical virginity, Religiosity

## Abstract

Previous studies have found that a large proportion of college students do not consider oral-genital contact as having “had sex.” In all studies, the questions posed were hypothetical. In the present study, university students were asked about their own personal sexual experiences. From a large pool of participants, two subgroups were identified: those who responded “No” to having “had sex” but responded “Yes” to having had oral-genital contact (No–Yes), and those who responded “Yes” to having had both sex and oral-genital contact (Yes–Yes). None of the participants in these two subgroups self-reported vaginal or anal intercourse. The No–Yes group was significantly higher in social desirability (*p* < .0005) as measured by the Marlowe-Crowne scale and was also significantly higher in religiosity (*p* < .01) as measured on a 7-point Likert scale. There was a modest correlation between level of religiosity and social desirability (*r* = .25, *p* < .01). It was concluded that many students who have had oral-genital contact but deny having had sex do so because of impression management, i.e., a desire to present themselves more positively. These results provide further evidence that social desirability responding is a serious problem for sex researchers, one that affects even the most basic questions about having had sex.

## Introduction

Due to the private nature of sexual behavior, most of what is known about it has relied on self-reported information. People’s responses will differ depending on what they regard to be “sex” or “had sex” and research has shown that opinions differ widely regarding the meaning of these terms. Several studies have found a hierarchy of agreement as to whether certain acts are regarded as sex (see Horowitz & Bedford, 2017). From highest agreement to least agreement, the hierarchy was vaginal intercourse, anal intercourse, oral-genital contact, manual-genital contact, and non-genital contact behaviors.

With regard to oral-genital contact, Sanders and Reinisch (1999) found that 60% of college students surveyed at a Midwest university in 1991 did not regard this behavior as “had sex.” Since then, surveys conducted on college campuses in the United States, Canada, Australia, and the United Kingdom have continued to find that a large proportion of heterosexual students do not regard oral-genital contact as having had sex (Bogart et al., 2000; Byers et al., 2009; Gute et al., 2008; Hans et al., 2010; Horowitz & Spicer, 2013; Pitts & Rahman, 2001; Randall & Byers, 2002; Richters & Song, 1999; Trotter & Alderson, 2007). The term “technical virgins” has been used by some researchers to refer to individuals who have had only oral-genital contact (not intercourse) but deny having had sex (Medley-Rath, 2007; Uecker et al., 2008). Although the percentage of college students who do not consider oral-genital contact as sex has decreased since 1991 (Hans et al., 2010), most studies still find that 25–44% of students believe this, particularly if oral-genital contact does not result in orgasm (Bogart et al., 2000; Byers et al., 2009; Randall & Byers, 2002; Trotter & Alderson, 2007).

Little is known about the personal characteristics of people who do not regard oral-genital contact as sex. Past sexual experience does not influence students’ beliefs as to whether oral-genital contact is considered as having had sex (Hans et al., 2010; Randall & Byers, 2002; Trotter & Alderson, 2007). Some studies found that women are less likely than men to regard oral-genital contact as sex (Bogart et al., 2000; Byers et al., 2009), but other studies found no gender differences (Hans et al., 2010; Horowitz & Spicer, 2013; Randall & Byers, 2002).

One possibility is that students’ self-reported sexual behaviors and attitudes are associated with social desirability, i.e., “the need of [individuals] to obtain approval by responding in a culturally appropriate manner” (Crowne & Marlowe, 1960: p. 353). Social desirability responding has been associated with many self-reported sexual behaviors (see King, 2022, for a review). The component of social desirability responding that is of most concern to sex researchers is called impression management, whereby a respondent knowingly does not tell the truth in order to present himself or herself positively (Paulhus, 1984). Impression management has been documented in sex surveys that relied on self-reports (e.g., Meston et al., 1998). As evidence that impression management may be a factor in some students’ denial of oral-genital contact as sex, a substantially greater percentage of people regard oral-genital contact as “had sex” if the question pertains to their partner’s behavior outside the relationship (Randall & Byers, 2002; Sewell & Strassberg, 2015).

One of the factors that may contribute to social desirability responding to people’s definitions of sex is level of religiosity. Religion is one of the main socializing agents for many teens and young adults (Penhollow et al., 2005). The more religious a college student is, the more likely that he or she will abstain from sexual intercourse (Sprecher & Treger, 2015). Studies find little difference among religions, but that it is the level of religiosity or spirituality that is important to conservative sexual behaviors and attitudes (Ahrold et al., 2011; de Visser et al., 2007). Higher levels of religiosity are associated with higher levels of sexual guilt (Woo et al., 2012). If social desirability is a factor, people will adjust their own definition of sex in order to avoid negative feelings about themselves after partaking in a particular type of sexual activity (Peterson & Muehlenhard, 2007). Thus, to avoid guilt, it is possible that individuals high in religiosity would deny that their oral-genital behaviors are considered as having sex.

In all previous studies that asked students if they considered oral-genital contact as “had sex,” the questions were hypothetical. Most used the same wording as Sanders and Reinisch (1999): “Would you say you ‘had sex’ if….” (e.g., Byers et al., 2009; Gute et al., 2008; Hans et al., 2010). Others used variations of this: e.g., “Which of the following activities count as ‘having sex with’…” (Richters & Song, 1999). In the present study, students were asked about their own personal sexual experiences and two specific subgroups who answered “Yes” to having engaged in oral-genital contact (but not vaginal or anal intercourse) were identified: those who answered “Yes” to having had sex with another person (Yes–Yes group) and those who answered “No” (No–Yes group). Thus, in terms of sexual behaviors, the two groups differed only with regard to acknowledging past oral-genital contact as having had sex. It was hypothesized that those students who denied sexual activity would be higher in both social desirability and religiosity.

## Method

### Participants

The study began with 1182 undergraduate students at the start of class in a human sexuality course at Clemson University during the Fall 2019 and Spring 2020 semesters. Participation was voluntary and students who did not wish to participate were told to hand in a blank questionnaire. Eighteen students chose not to participate and the data for nine others were eliminated because they did not answer all the questions. Among the 1155 students who completed the questionnaire, nearly three-fourths (74.6%) were women and 25.4% were men. The large female/male ratio is typical of university human sexuality courses (King et al., 2020).

A large initial sample was necessary because it was anticipated that a relatively small percentage of students in this age group would be in the two subgroups of interest. Most college students’ sexual experience will have already included vaginal and/or anal intercourse, while others will not yet have engaged in either sexual intercourse or oral-genital contact. In the final sample, 847 students (73.3%) self-reported having “had sex” that included vaginal intercourse (and often oral-genital contact and/or anal intercourse as well), and 194 students (16.8%) who answered “No” to all four questions about sexual behavior. The present study focused on two other subgroups: (1) No–Yes group: 77 students (65 women, 12 men; 73 white, 2 Hispanics, and 2 mixed race; mean age = 19.4) who answered “No” to “Have you had sex with another person?” and having engaged in vaginal or anal intercourse, but answered “Yes” to having had oral-genital contact, and (2) Yes–Yes group: 30 students (19 women, 11 men; 23 white, 2 African Americans, 2 Hispanics, and 3 Asian-American; mean age = 20.1) who answered “Yes” to having had sex and also to having engaged in oral-genital contact, but “No” to vaginal and anal intercourse.

### Measures

The paper-and-pencil, anonymously-answered questionnaire included demographic questions, the Marlowe-Crowne scale for social desirability (Crowne & Marlowe, 1960), and the question “How religious are you?” answered with a 7-point Likert scale (0 = not at all to 6 = very). The Marlowe-Crowne scale has 33 True–False questions (none pertaining to sex) that provide an overall assessment of an individual’s desire to respond in a culturally approved manner (cited over 13,000 times). Example questions: “I am always courteous, even to people who are disagreeable”; “No matter who I’m talking to, I’m always a good listener”; and “I’m always willing to admit it when I make a mistake.”

There were four questions about sexual behavior: (1) “Have you had sex with another person?”; (2) “Have you participated in vaginal intercourse (penile-vaginal)?”; (3) “Have you had mouth-genital or genital-mouth contact with another person?”; and (4) “Have you had anal intercourse with another person?” These four questions were placed in that order and spaced equally throughout the Marlowe-Crowne scale. Asking participants whether they had personally “had sex” or engaged in oral-genital contact (rather than asking hypothetical questions) allowed the authors to ascertain whether there was an association with their level of social desirability. The instructor did not participate in the distribution or collection of the questionnaires. The study was approved by the university’s IRB.

Differences between the two groups were evaluated by *t*-tests. Degree of association between social desirability and religiosity was evaluated with Pearson’s *r*.

## Results

The raw data are shared in a data repository: osf.io/enm57/. The mean (± SD) Marlowe-Crowne scores for the No–Yes group (14.99 ± 4.79) were significantly higher than the mean (11.47 ± 3.59) for the Yes–Yes group (*t* = 3.61, df = 105, *p* < 0.0005). See Table 1. The effect size (*g* = 0.78) was close to what Cohen (1988) considered to be a large effect (0.8). For level of religiosity, the mean score was 4.22 ± 1.64 for the No–Yes group and 3.17 ± 1.71 for the Yes–Yes group (*t* = 2.92, df = 105, *p* < 0.01, *g* = 0.63). There was a significant correlation between strength of religiosity and Marlowe-Crowne scores (*r* = 0.25, df = 106, *p* < 0.01). There was a significant gender difference between the No–Yes group and the Yes–Yes group, with women comprising a larger proportion of the former (*χ*^2^ = 5.68, df = 1, *p* < 0.05). However, the effect size (phi = 0.23) was small (Cohen, 1988).

**Table 1:** Social desirability scores (M ± SD) and self-reported level of religiosity for college students who answered “No” to having had sex and “Yes” to having had oral-genital contact, and for students who answered “Yes” to both

	No–Yes group (*n* = 77)	Yes–Yes group (*n* = 30)
Marlowe-Crowne scores	14.99 ± 4.79	11.47 ± 3.59
Religiosity (0 to 6)	4.22 ± 1.64	3.17 ± 1.71

## Discussion

Participants in both the No–Yes and Yes–Yes groups admitted to having had oral-genital contact and differed only on their answer to whether they had “had sex” (none said that they had engaged in vaginal or anal intercourse). The mean Marlowe-Crowne score for the sample that answered “No” to having had sex but “Yes” to having engaged in oral-genital contact was significantly greater than for the Yes–Yes sample. These results show that individuals who deny having engaged in sex but have had oral-genital relations are higher in social desirability than those individuals who have had oral-genital sexual relations and answer “Yes” when asked if they have had sex. Thus, individuals in the No–Yes group were more likely to respond in a manner considered culturally appropriate (Crowne & Marlowe, 1960). The gender difference that was found (i.e., proportionately more women in the No–Yes group) supports this. Studies find that many college students are influenced by traditional sexual scripts, including the attitude that women are sexual gatekeepers (e.g., Hirsch et al., 2019; Jozkowski & Peterson, 2013).

It has long been recognized that the generalizability of studies of self-reported sexual behaviors is limited by the possibility of volunteer bias, i.e., that individuals who volunteer for the studies differ in important ways from individuals who do not (Bogaert, 1996; Strassberg & Lowe, 1995). Even among individuals who volunteer, social desirability responding may further decrease the generalizability of results. Social desirability bias has been found to be pervasive and often extreme in health research and other fields in which researchers can verify self-reported behaviors (e.g., see Archer et al., 2015; Ioannidis, 2013). Although sex researchers generally lack a gold standard by which to compare the truthfulness of self-reported behaviors, there is increasing evidence that social desirability bias is as common as in other fields. Social desirability responding has been found to be associated with self-reported information for HIV high-risk behaviors (Gibson et al., 1999; Latkin & Vlahov, 1998), heterosexual anal intercourse (Bolling & Voeller, 1987), extramarital affairs (Zapien, 2017), use of condoms (Rao et al., 2017), exposure to pornography (Rasmussen et al., 2018), and penis size (King et al., 2019). A recent study found that one factor contributing to men’s reporting more opposite-sex partners than women is misreporting due to perceived gendered norms (Mitchell et al., 2019). The present study shows that even some individuals’ definition of “sex” or “had sex” is influenced by social desirability. Of particular interest to the present study, Meston et al. (1998) found a modest but significant correlation (0.18) between virginity status for females (scored as a dichotomous variable) and impression management.

The modest correlation between religiosity and social desirability suggests that high religiosity is associated with social desirability responding by some students. Many young people feel guilty for sexual attitudes that go against religious and moral ideologies (Olson et al., 2014; Woo et al., 2012) and present themselves as “technical virgins” if they have had only oral-genital contact (Medley-Rath, 2007; Uecker et al., 2008) in order to align with their church’s teachings and “stay pure” (Brückner & Bearman, 2005). Peterson and Muehlenhard (2007) found that some women chose not to define oral-genital contact as sex because “it allowed them to maintain their identity as virgins” (p. 262).

Not all of the results for the No–Yes group can be attributed to greater religiosity (*r*^2^ was only 0.06). Thirteen of the 77 participants in the No–Yes group rated themselves as having low religiosity (three = 0, four = 1, six = 2). Obviously, other factors besides religiosity influenced the responses for these individuals and contributed to the variance. In a recent study, a modest correlation was found between self-reported use of pornography and social desirability bias, but there was no relationship between social desirability and religiosity (Rasmussen et al., 2018).

Meston et al. (1998) found that impression management was moderately associated with conscientiousness in both men and women, and with agreeableness in women. Uecker et al. (2008) found that many people who engaged in oral-genital contact but abstained from sexual intercourse were motivated more by a desire to avoid pregnancy and sexually transmitted infections than by religiosity. Others have found similar reasons (Michels et al., 2005), but this does not explain why many of these individuals would deny that their engaging in oral-genital contact was sex. Social desirability responding can account for the discrepancy.

The reasons for social desirability responding by some individuals regarding their oral-genital behavior may not be fully understood, but it is important to recognize that it has occurred. The present study provides further evidence that sex researchers must be explicit when asking people about their sexual experiences. However, researchers need to be aware that even when questions about sexual behaviors are explicit and answered anonymously social desirability responding is common (see King, 2021, for a review). Researchers who rely on participants’ self-reported sexual behaviors need to address social desirability responding when designing their studies and reporting results.

### Limitations

The study used a convenience sample drawn from a single university. Previous studies have found ethnic differences in sexual attitudes (e.g., Ahrold & Meston, 2010). However, the number of ethnic minority students at this university is small and the number of minority participants in the final samples was too small for meaningful comparisons of attitudes about oral-genital contact as sex. Thus, the results cannot be generalized to other ethnic/racial or age groups. The same is true of previous studies of college students who did not regard oral-genital contact as sex. Future research should include a wider variety of participants.

## Declarations

### Data Availability

The raw data may be found at osf.io/enm57/
